# The Universal Plausibility Metric (UPM) & Principle (UPP)

**DOI:** 10.1186/1742-4682-6-27

**Published:** 2009-12-03

**Authors:** David L Abel

**Affiliations:** 1 Department of ProtoBioCybernetics/ProtoBioSemiotics, The Gene Emergence Project of The Origin of Life Science Foundation, Inc, 113-120 Hedgewood Dr Greenbelt, MD 20770-1610, USA

## Abstract

**Background:**

Mere possibility is not an adequate basis for asserting scientific plausibility. A precisely defined universal bound is needed beyond which the assertion of *plausibility*, particularly in life-origin models, can be considered operationally falsified. But can something so seemingly relative and subjective as plausibility ever be quantified? Amazingly, the answer is, "Yes." A method of objectively measuring the plausibility of any chance hypothesis (The Universal Plausibility Metric [UPM]) is presented. A numerical inequality is also provided whereby any chance hypothesis can be definitively falsified when its UPM metric of ξ is < 1 (The Universal Plausibility Principle [UPP]). Both UPM and UPP pre-exist and are independent of any experimental design and data set.

**Conclusion:**

No low-probability hypothetical plausibility assertion should survive peer-review without subjection to the UPP inequality standard of formal falsification (ξ < 1).

## The seemingly subjective liquidity of "plausibility"

Are there any *objective *standards that could be applied to evaluate the seemingly subjective notion of *plausibility*? Can something so psychologically relative as plausibility ever be quantified?

Our skepticism about defining a precise, objective Universal Plausibility Metric (UPM) stems from a healthy realization of our finiteness [1], subjectivity [2], presuppositional biases [3,4], and epistemological problem [5]. We are rightly wary of absolutism. The very nature of probability theory emphasizes gray-scales more than the black and white extremes of p = 0 or 1.0. Our problem is that extremely low probabilities can only asymptotically approach impossibility. An extremely unlikely event's probability always remains at least slightly > 0. No matter how many orders of magnitude is the negative exponent of an event's probability, that event or scenario technically cannot be considered impossible. Not even a Universal Probability Bound [6-8] seems to establish absolute theoretical impossibility. The fanatical pursuit of absoluteness by finite subjective knowers is considered counterproductive in post modern science. Open-mindedness to all possibilities is encouraged [9].

But at some point our reluctance to exclude *any *possibility becomes stultifying to operational science [10]. Falsification is critical to narrowing down the list of serious possibilities [11]. Almost all hypotheses are possible. Few of them wind up being helpful and scientifically productive. Just because a hypothesis is possible should not grant that hypothesis scientific respectability. More attention to the concept of "infeasibility" has been suggested [12]. Millions of dollars in astrobiology grant money have been wasted on scenarios that are possible, but plausibly bankrupt. The question for scientific methodology should *not *be, "Is this scenario possible?" The question should be, "Is this possibility a *plausible *scientific hypothesis?" One chance in 10^200 ^is theoretically possible, but given maximum cosmic probabilistic resources, such a possibility is hardly plausible. With funding resources rapidly drying up, science needs a foundational principle by which to falsify a myriad of theoretical possibilities that are not worthy of serious scientific consideration and modeling.

Proving a theory is considered technically unachievable [11]. Few bench scientists realize that falsification has also been shown by philosophers of science to be at best technically suspect [13]. Nevertheless, operational science has no choice but to proceed primarily by a process of elimination through practical falsification of competing models and theories.

Which model or theory best corresponds to the data? [[14] (pg. 32-98)] [8]. Which model or theory best predicts future interactions? Answering these questions is made easier by eliminating implausible possibilities from the list of theoretical possibilities. Great care must be taken at this point, especially given the many non intuitive aspects of scientifically addressable reality. But operational science must proceed on the basis of best-thus-far tentative knowledge. The human epistemological problem is quite real. But we cannot allow it to paralyze scientific inquiry.

If it is true that we cannot *know *anything for certain, then we have all the more reason to proceed on the basis of the greatest "*plausibility *of belief" [15-19]. If human mental constructions cannot be equated with objective reality, we are all the more justified in pursuing the greatest likelihood of correspondence of our knowledge to the object of that knowledge--presumed ontological being itself. Can we prove that objectivity exists outside of our minds? No. Does that establish that objectivity does not exist outside of our minds? No again. Science makes its best progress based on the axioms that 1) an objective reality independent of our minds does exist, and 2) scientists' collective knowledge can progressively correspond to that objective reality. The human epistemological problem is kept in its proper place through a) double-blind studies, b) groups of independent investigators all repeating the same experiment, c) prediction fulfillments, and d) the application of pristine logic (taking linguistic fuzziness into account), and e) the competition of various human ideas for best correspondence to repeated independent observations.

The physical law equations and the deductive system of mathematical rules that govern the manipulations of those equations are all formally absolute. But the axioms from which formal logic theory flows, and the decision of when to consider mathematical equations universal "laws" are not absolute. Acceptance of mathematical axioms is hypothetico-deductively relative. Acceptance of physical laws is inductively relative. The *pursuit *of correspondence between presumed objective reality and our knowledge of objective reality is laudable in science. But not even the axioms of mathematics or the laws of physics can be viewed as absolute. Science of necessity proceeds tentatively on the basis of best-thus-far subjective knowledge. At some admittedly relative point, the scientific community agrees by consensus to declare certain formal equations to be reliable descriptors and predictors of future physicodynamic interactions. Eventually the correspondence level between our knowledge and our repeated observations of presumed objective reality is considered *adequate *to make a tentative *commitment *to the veracity of an axiom or universal law until they are proven otherwise.

The same standard should apply in falsifying ridiculously implausible life-origin assertions. Combinatorial imaginings and hypothetical scenarios can be endlessly argued simply on the grounds that they are theoretically possible. But there is a point beyond which arguing the plausibility of an absurdly low probability becomes *operationally *counterproductive. That point can actually be quantified for universal application to all fields of science, not just astrobiology. Quantification of a UPM and application of the UPP inequality test to that specific UPM provides for definitive, unequivocal falsification of scientifically unhelpful and functionally useless hypotheses. When the UPP is violated, declaring falsification of that highly implausible notion is just as justified as the firm commitment we make to any mathematical axiom or physical "law" of motion.

## Universal Probability Bounds

"Statistical prohibitiveness" in probability theory and the physical sciences has remained a nebulous concept for far too long. The importance of probabilistic resources as a context for consideration of extremely low probabilities has been previously emphasized [[20] (pg. 13-17)] [6-8,21]. Statistical prohibitiveness cannot be established by an exceedingly low probability alone [6]. Rejection regions and probability bounds need to be established independent of (preferably prior to) experimentation in any experimental design. But the setting of these zones and bounds is all too relative and variable from one experimental design to the next. In the end, however, probability is not the critical issue. The plausibility of hypotheses is the real issue. Even more important is the question of whether we can ever operationally falsify a preposterous but theoretically possible hypothesis.

The Universal Probability Bound (UPB) [6,7] quantifies the maximum cosmic probabilistic resources (Ω, upper case omega) as the context of evaluation of any extremely low probability event. Ω corresponds to the maximum number of possible probabilistic trials (quantum transitions or physicochemical interactions) that could have occurred in cosmic history. The value of Ω is calculated by taking the product of three factors:

1) The number of seconds that have elapsed since the Big Bang (10^17^) assumes a cosmic age of around 14 billions years. 60 sec/min × 60 min/hr × 24 hrs/day × 365 days per year × 14 billion years = 4.4 × 10^17 ^seconds since the Big Bang.

2) The number of possible quantum events/transitions per second is derived from the amount of time it takes for light to traverse the minimum unit of distance. The minimum unit of distance (a quantum of space) is Planck length (10^-33 ^centimeters). The minimum amount of time required for light to traverse the Plank length is Plank time (10^-43 ^seconds) [[6-8], pg 215-217]. Thus a maximum of 10^43 ^quantum transitions can take place per second. Since 10^17 ^seconds have elapsed since the Big Bang, the number of possible quantum transitions since the Big Bang would be 10^43 ^× 10^17 ^= 10^60^.

3) Sir Arthur Eddington's estimate of the number of protons, neutrons and electrons in the observable cosmos (10^80^) [22] has been widely respected throughout the scientific literature for decades now.

Some estimates of the total number of elementary particles have been slightly higher. The Universe is 95 billion light years (30 gigaparsecs) across. We can convert this to cubic centimeters using the equation for the volume of a sphere (5 × 10^86 ^cc). If we multiply this times 500 particles (100 neutrinos and 400 photons) per cc, we would get 2.5 × 10^89 ^elementary particles in the visible universe.

A Universal Probability Bound could therefore be calculated by the product of these three factors: 10^17 ^× 10^43 ^× 10^80 ^= 10^140^

If the highest estimate of the number of elementary particles in the Universe is used (e.g., 10^89^), the UPB would be 10^149^.

The UPB's discussed above are *the highest calculated universal probability bounds ever published by many orders of magnitude *[7,8,12]. They are the most permissive of (favorable to) extremely low-probability plausibility assertions in print [6] [[8] (pg. 216-217)]. All other proposed metrics of probabilistic resources are far less permissive of low-probability chance-hypothesis plausibility assertions. Emile Borel's limit of cosmic probabilistic resources was only 10^50 ^[[23] (pg. 28-30)]. Borel based this probability bound in part on the product of the number of observable stars (10^9^) times the number of possible human observations that could be made on those stars (10^20^). Physicist Bret Van de Sande at the University of Pittsburgh calculates a UPB of 2.6 × 10^92 ^[8,24]. Cryptographers tend to use the figure of 10^94 ^computational steps as the resource limit to any cryptosystem's decryption [25]. MIT's Seth Lloyd has calculated that the universe could not have performed more than 10^120 ^bit operations in its history [26].

Here we must point out that a discussion of the number of cybernetic or cryptographic "operations" is totally inappropriate in determining a prebiotic UPB. Probabilistic combinatorics has nothing to do with "operations." Operations involve choice contingency [27-29]. Bits are "Yes/No" question *opportunities *[[30] (pg. 66)], each of which could potentially reduce the total number of combinatorial possibilities (2^NH ^possible biopolymers: see Appendix 1) by half. But of course asking the right question and getting an answer is not a spontaneous physicochemical phenomenon describable by mere probabilistic uncertainty measures [31-33]. Any binary "operation" involves a bona fide decision node [34-36]. An operation is a *formal choice-based function*. Shannon uncertainty measures do not apply to specific choices [37-39]. Bits measure only the number of non distinct, generic, *potential *binary choices, not actual specific choices [37]. Inanimate nature cannot ask questions, get answers, and exercise choice contingency at decision nodes in response to those answers. Inanimate nature cannot optimize algorithms, compute, pursue formal function, or program configurable switches to achieve integration and shortcuts to formal utility [28]. Cybernetic operations therefore have no bearing whatever in determining universal probability bounds for chance hypotheses.

Agreement on a sensible UPB in advance of (or at least totally independent of) any specific hypothesis, suggested scenario, or theory of mechanism is critical to experimental design. No known empirical or rational considerations exist to preclude acceptance of the above UPB. The only exceptions in print seem to come from investigators who argue that the above UPB is *too permissive *of the chance hypothesis [8,12]. Faddish acceptance prevails of hypothetical scenarios of extremely low probability simply because they are in vogue and are theoretically possible. Not only a UPB is needed, but a fixed universal mathematical standard of *plausibility *is needed. This is especially true for complex hypothetical scenarios involving joint and/or conditional probabilities. Many imaginative hypothetical scenarios propose constellations of highly cooperative events that are theorized to self-organize into holistic formal schemes. Whether joint, conditional or independent, multiple probabilities must be factored into an overall plausibility metric. In addition, a universal plausibility bound is needed to eliminate overly imaginative fantasies from consideration for the best inference to causation.

## The Universal Plausibility Metric (UPM)

To be able to definitively falsify ridiculously implausible hypotheses, we need first a Universal *Plausibility *Metric (UPM) to assign a numerical plausibility value to each proposed hypothetical scenario. Second, a Universal Plausibility Principle (UPP) inequality is needed as plausibility bound of this measurement for falsification evaluation. We need a cut-off point beyond which no extremely low probability scenario can be considered a "*scientifically respectable*" possibility. What is needed more than a probability bound is a *plausibility *bound. Any "possibility" that exceeds the ability of its probabilistic resources to generate should immediately be considered a "*functional non possibility*," and therefore an implausible scenario. While it may not be a theoretically absolute impossibility, if it exceeds its probabilistic resources, it is a gross understatement to declare that such a proposed scenario is simply not worth the expenditure of serious scientific consideration, pursuit, and resources. Every field of scientific investigation, not just biophysics and life-origin science, needs the application of the same independent test of credibility to judge the plausibility of its hypothetical events and scenarios. The application of this standard should be an integral component of the scientific method itself for all fields of scientific inquiry.

To arrive at the UPM, we begin with the maximum available probabilistic resources discussed above (Ω, upper case Omega) [6,7]. But Ω could be considered from a quantum or a classical molecular/chemical perspective. Thus this paper proposes that the Ω quantification be broken down first according to the Level (L) or perspective of physicodynamic analysis (^L^Ω), where the perspective at the quantum level is represented by the superscript "q" (^q^Ω) and the perspective at the classical level is represented by "c" (^c^Ω). Each represents the maximum probabilistic resources available at each level of physical activity being evaluated, with the total number of quantum transitions being much larger than the total number of "ordinary" chemical reactions since the Big Bang.

Second, the maximum probabilistic resources ^L^Ω (^q^Ω for the quantum level and ^c^Ω for classical molecular/chemical level) can be broken down even further according to the astronomical subset being addressed using the general subscript "A" for Astronomical: ^L^Ω_A _(representing both ^q^Ω_A _and ^c^Ω_A_). The maximum probabilistic resources can then be measured for each of the four different specific environments of each ^L^Ω, where the general subscript A is specifically enumerated with "u" for universe, "g" for our galaxy, "s" for our solar system, and "e" for earth:

To include meteorite and panspermia inoculations in the earth metrics, we use the Solar System metrics ^L^Ω_s _(^q^Ω_s _and ^c^Ω_s_).

As examples, for quantification of the maximum probabilistic resources at the quantum level for the astronomical subset of our galactic phase space, we would use the ^q^Ω_g _metric. For quantification of the maximum probabilistic resources at the ordinary classical molecular/chemical reaction level in our solar system, we would use the ^c^Ω_s _metric.

The most permissive UPM possible would employ the probabilistic resources symbolized by ^q^Ω_u _where both the quantum level perspective and the entire universe are considered.

The sub division between the ^L^Ω_A _for the quantum perspective (quantified by ^q^Ω_A_) and that for the classical molecular/chemical perspective (quantified by ^c^Ω_A_), however, is often not as clear and precise as we might wish. Crossovers frequently occur. This is particularly true where quantum events have direct bearing on "ordinary" chemical reactions in the "everyday" classical world. If we are going to err in evaluating the plausibility of any hypothetical scenario, let us err in favor of maximizing the probabilistic resources of ^L^Ω_A_. In cases where quantum factors seem to directly affect chemical reactions, we would want to use the four quantum level metrics of ^q^Ω_A _(^q^Ω_u_, ^q^Ω_g_, ^q^Ω_s _and ^q^Ω_e_) to preserve the plausibility of the lowest-probability explanations.

## Quantification of the Universal Plausibility Metric (UPM)

The computed Universal Plausibility Metric (UPM) objectively quantifies the level of plausibility of any chance hypothesis or theory. The UPM employs the symbol ξ (Xi, pronounced *zai *in American English, *sai *in UK English, *ksi *in modern Greek) to represent the computed UPM according to the following equation:(1)

where *f *represents the number of functional objects/events/scenarios that are known to occur out of all possible combinations (lower case omega, ω) (e.g., the number [*f*] of functional protein family members of varying sequence known to occur out of sequence space [ω]), and ^L^Ω_A _(upper case Omega, Ω) represents the total probabilistic resources for any particular probabilistic context. The "L" superscript context of Ω describes which perspective of analysis, whether quantum (q) or a classical (c), and the "A" subscript context of Ω enumerates which subset of astronomical phase space is being evaluated: "u" for universe, "g" for our galaxy, "s" for our solar system, and "e" for earth. Note that the basic generic UPM (ξ) equation's form remains constant despite changes in the variables of levels of perspective (L: whether q or c) and astronomic subsets (A: whether u, g, s, or e).

The calculations of probabilistic resources in ^L^Ω_A _can be found in Appendix 2. Note that the upper and lower case omega symbols used in this equation are *case sensitive *and each represents a completely different phase space.

The UPM from both the quantum (^q^Ω_A_) and classical molecular/chemical (^c^Ω_A_) perspectives/levels can be quantified by Equation 1. This equation incorporates *the number of possible transitions or physical interactions that could have occurred since the Big Bang*. Maximum quantum-perspective probabilistic resources ^q^Ω_u _were enumerated above in the discussion of a UPB [6,7] [[8] (pg. 215-217)]. Here we use basically the same approach with slight modifications to the factored probabilistic resources that comprise Ω.

Let us address the quantum level perspective (q) first for the entire universe (u) followed by three astronomical subsets: our galaxy (g), our solar system (s) and earth (e).

Since approximately 10^17 ^seconds have elapsed since the Big Bang, we factor that total time into the following calculations of quantum perspective probabilistic resource measures. Note that the difference between the age of the earth and the age of the cosmos is only a factor of 3. A factor of 3 is rather negligible at the high order of magnitude of 10^17 ^seconds since the Big Bang (versus age of the earth). Thus, 10^17 ^seconds is used for all three astronomical subsets:

These above limits of probabilistic resources exist within the only known universe that we can repeatedly observe--the only universe that is scientifically addressable. Wild metaphysical claims of an infinite number of cosmoses may be fine for cosmological imagination, religious belief, or superstition. But such conjecturing has no place in hard science. Such claims cannot be empirically investigated, and they certainly cannot be falsified. They violate Ockham's (Occam's) Razor [40]. No prediction fulfillments are realizable. They are therefore nothing more than blind beliefs that are totally inappropriate in peer-reviewed scientific literature. Such cosmological conjectures are far closer to metaphysical or philosophic enterprises than they are to bench science.

From a more classical perspective at the level of ordinary molecular/chemical reactions, we will again provide metrics first for the entire universe (u) followed by three astronomical subsets, our galaxy (g), our solar system (s) and earth (e).

The classical molecular/chemical perspective makes two primary changes from the quantum perspective. With the classical perspective, *the number of atoms *rather than the number of protons, neutrons and electrons is used. In addition, *the total number of classical chemical reactions that could have taken place since the Big Bang *is used rather than transitions related to cubic light-Planck's. The shortest time any transition requires before a chemical reaction can take place is 10 femtoseconds [41-46]. A femtosecond is 10^-15 ^seconds. Complete chemical reactions, however, rarely take place faster than the picosecond range (10^-12 ^secs). Most biochemical reactions, even with highly sophisticated enzymatic catalysis, take place no faster than the nano (10^-9^) and usually the micro (10^-6^) range. To be exceedingly generous (perhaps overly permissive of the capabilities of the chance hypothesis), we shall use 100 femtoseconds as the shortest chemical reaction time. 100 femtoseconds is 10^-13 ^seconds. Thus 10^13 ^simple and fastest chemical reactions could conceivably take place per second in the best of theoretical pipe-dream scenarios. The four ^c^Ω_A _measures are as follows:

      


Remember that ^L^Ω_e _excludes meteorite and panspermia inoculations. To include meteorite and panspermia inoculations, we use the metric for our solar system ^c^Ω_s_.

These maximum metrics of the limit of probabilistic resources are based on the best-thus-far estimates of a large body of collective scientific investigations. We can expect slight variations up or down of our best guesses of the number of elementary particles in the universe, for example. But the basic formula presented as the Universal Plausibility Metric (PM) will never change. The Universal Plausibility Principle (UPP) inequality presented below is also immutable and worthy of law-like status. It affords the ability to objectively once and for all falsify not just highly improbable, but ridiculously implausible scenarios. Slight adjustments to the factors that contribute to the value of each ^L^Ω_A _are straightforward and easy for the scientific community to update through time.

Most chemical reactions take longer by many orders of magnitude than what these exceedingly liberal maximum probabilistic resources allow. Biochemical reactions can take years to occur in the absence of highly sophisticated protein enzymes not present in a prebiotic environment. Even humanly engineered ribozymes rarely catalyze reactions by an enhancement rate of more than 10^5 ^[47-51]. Thus the use of the fastest rate known for any complete chemical reaction (100 femtoseconds) seems to be the most liberal/forgiving probability bound that could possibly be incorporated into the classical chemical probabilistic resource perspective ^c^Ω_A_. For this reason, we should be all the more ruthless in applying the UPP test of falsification presented below to seemingly "far-out" metaphysical hypotheses that have no place in responsible science.

## Falsification using The Universal Plausibility Principle (UPP)

The Universal Plausibility Principle (UPP) states that *definitive operational falsification *of any chance hypothesis is provided by the inequality of:

This definitive operational falsification holds for hypotheses, theories, models, or scenarios at any level of perspective (q or c) and for any astronomical subset (u, g, s, and e). The UPP inequality's falsification is valid whether the hypothesized event is singular or compound, independent or conditional. Great care must be taken, however, to eliminate errors in the calculation of complex probabilities. Every aspect of the hypothesized scenario must have its probabilistic components factored into the one probability (*p*) that is used in the UPM (See equation 2 below). Many such combinatorial possibilities are joint or conditional. It is not sufficient to factor only the probabilities of each reactant's formation, for example, while omitting the probabilistic aspects of each reactant being presented at the same place and time, becoming available in the required reaction order, or being able to react at all (activated vs. not activated). Other factors must be included in the calculation of probabilities: optical isomers, non-peptide bond formation, many non biological amino acids that also react [8]. The exact calculation of such probabilities is often not straightforward. But in many cases it becomes readily apparent that whatever the exact multi-factored calculation, the probability "*p*" of the entire scenario easily crosses the plausibility bound provided by the UPP inequality. This provides a definitive objective standard of falsification. When ξ < 1, immediately the notion should be considered "not a *scientifically plausible *possibility." A ξ value < 1 should serve as an unequivocal operational falsification of that hypothesis. The hypothetical scenario or theory generating that ξ metric should be excluded from the differential list of possible causes. The hypothetical notion should be declared to be outside the bounds of scientific respectability. It should be flatly rejected as the equivalent of superstition.

*f*/ω in Equation 1 is in effect the probability of a particular *functional *event or object occurring out of all possible combinations. Take for example an RNA-World model. 23 different functional ribozymes in the same family might arise out of 10^15 ^stochastic ensembles of 50-mer RNAs. This would reduce to a probability *p *of roughly 10^-14 ^of getting a stochastic ensemble that manifested some degree of that ribozyme family's function.

Thus *f*/ω in Equation 1 reduces to the equivalent of a probability *p*:(2)

where "*p*" represents an extremely low probability of any chance hypothesis that is *asserted to be plausible *given ^L^Ω_A _probabilistic resources, in this particular case ^c^Ω_e _probabilistic resources.

As examples of attempts to falsify, suppose we have three different chance hypotheses, each with its own low probability (*p*), all being evaluated from the quantum perspective at the astronomical level of the entire universe (^q^Ω_u_). Given the three different probabilities (p) provided below, the applied UPP inequality for each ξ = p ^q^Ω_u _of each hypothetical scenario would establish definitive operational falsification for one of these three hypothetical scenarios, and fail to falsify two others:

Let us quantify an example of the use of the UPM and UPP to attempt falsification of a chance hypothetical scenario:

Suppose 10^3 ^*biofunctional *polymeric sequences of monomers (*f*) exist out of 10^17 ^possible sequences in sequence space (ω) all of the same number (*N*) of monomers. That would correspond to one chance in 10^14 ^of getting a functional sequence by chance (*p *= 10^3^/10^17 ^= 1/10^14 ^= 10^-14 ^of getting a functional sequence). If we were measuring the UPM from the perspective of a classical chemical view on earth over the last 5 billion years (^c^Ω_e _= 10^70^), we would use the following UPM equation (#1 above) with substituted values:

Since ξ > 1, this particular chance hypothesis is shown unequivocally to be plausible and worthy of further scientific investigation.

As one of the reviewers of this manuscript has pointed out, however, we might find the sequence space ω, and therefore the probability space *f*/ω, to be radically different for abiogenesis than for general physico-chemical reactions. The sequence space ω must include factors such as heterochirality, unwanted non-peptide-bond formation, and the large number of non biological amino acids present in any prebiotic environment [8,12]. This greatly increases ω, and would tend to substantially reduce the probability *p *of naturalistic abiogenesis. Spontaneously biofunctional stochastic ensemble formation was found to be only 1 in 10^64 ^when TEM-1 β-lactamase's working domain of around 150 amino acids was used as a model [52]. Function was related to the hydropathic signature necessary for proper folding (tertiary structure). The ability to confer any relative degree of beta-lactam penicillin-like antibiotic resistance to bacteria was considered to define "biofunctional" in this study. Axe further measured the probability of a random 150-residue primary structure producing *any *short protein, despite many allowable monomeric substitutions, to be 10^-74^. This probability is an example of a scientifically determined *p *that should be incorporated into any determination of the UPM in abiogenesis models.

## Don't multiverse models undermine The UPP?

Multiverse models imagine that our universe is only one of perhaps countless parallel universes [53-55]. Appeals to the Multiverse worldview are becoming more popular in life-origin research as the statistical prohibitiveness of spontaneous generation becomes more incontrovertible in a finite universe [56-58]. The term "notion," however, is more appropriate to refer to multiverse speculation than "theory." The idea of multiple parallel universes cannot legitimately qualify as a testable scientific hypothesis, let alone a mature theory. Entertaining multiverse "thought experiments" almost immediately takes us beyond the domain of responsible science into the realm of pure metaphysical belief and conjecture. The dogma is literally "beyond physics and astronomy," the very meaning of the word "metaphysical."

The notion of multiverse has no observational support, let alone repeated observations. Empirical justification is completely lacking. It has no testability: no falsification potential exists. If provides no prediction fulfillments. The non parsimonious construct of multiverse grossly violates the principle of Ockham's (Occam's) Razor [40]. No logical inference seems apparent to support the strained belief other than a perceived need to rationalize what we know is statistically prohibitive in the only universe that we *do *experience. Multiverse fantasies tend to constitute a back-door fire escape for when our models hit insurmountable roadblocks in the observable cosmos. When none of the facts fit our favorite model, we conveniently create imaginary extra universes that are more accommodating. This is not science. Science is interested in falsification within the only universe that science can address. Science cannot operate within mysticism, blind belief, or superstition. A multiverse may be fine for theoretical metaphysical models. But no justification exists for inclusion of this "dream world" in the sciences of physics and astronomy.

It could be argued that multiverse notions arose only in response to the severe time and space constraints arising out of Hawking, Ellis and Penrose's singularity theorems [59-61]. Solutions in general relativity involve singularities wherein matter is compressed to a point in space and light rays originate from a curvature. These theorems place severe limits on time and space since the Big Bang. Many of the prior assumptions of limitless time and sample space in naturalistic models were eliminated by the demonstration that time and space in the cosmos are quite finite, not infinite. For instance, we only have 10^17^-10^18 ^seconds at most to work with in any responsible cosmological universe model since the Big Bang. Glansdorff makes the point, "Conjectures about emergence of life in an infinite multiverse should not confuse probability with possibility." [62]

Even if multiple physical cosmoses existed, it is a logically sound deduction that linear digital genetic instructions using a representational material symbol system (MSS) [63] cannot be programmed by the chance and/or fixed laws of physicodynamics [27-29,32,33,36-39,64,65]. This fact is not only true of the physical universe, but would be just as true in any imagined physical multiverse. Physicality cannot generate non physical Prescriptive Information (PI) [29]. Physicodynamics cannot practice formalisms (The Cybernetic Cut) [27,34]. Constraints cannot exercise formal control unless those constraints are themselves chosen to achieve formal function [28] ("Constraints vs. Controls" currently in peer review). Environmental selection cannot select at the genetic level of arbitrary symbol sequencing (e.g., the polymerization of nucleotides and codons). (The GS Principle [Genetic Selection Principle]) [36,64]. Polymeric syntax (sequencing; primary structure) prescribes future (potential; not-yet-existent) folding and formal function of small RNAs and DNA. Symbol systems and configurable switch-settings can only be programmed with choice contingency, not chance contingency or fixed law, if non trivial coordination and formal organization are expected [29,38]. The all-important determinative sequencing of monomers is completed with rigid covalent bonds before any transcription, translation, or three-dimensional folding begins. Thus, imagining multiple physical universes or infinite time does not solve the problem of the origin of *formal *(non physical) biocybernetics and biosemiosis using a linear digital representational symbol system. The source of Prescriptive Information (PI) [29,35] in a metaphysically presupposed material-only world is closely related to the problem of gene emergence from physicodynamics alone. The latter hurdles remain the number-one enigmas of life-origin research [66].

The main subconscious motivation behind multiverse conjecture seems to be, "Multiverse models can do anything we want them to do to make our models work for us." We can argue Multiverse models ad infinitum because their potential is limitless. The notion of Multiverse has great appeal because it can explain everything (and therefore nothing). Multiverse models are beyond scientific critique, falsification, and prediction fulfillment verification. They are purely metaphysical.

Multiverse imaginings, therefore, offer no scientific threat whatever to the universality of the UPM and UPP in the only cosmic reality that science knows and investigates.

## Conclusion

Mere possibility is not an adequate basis for asserting scientific plausibility. Indeed, the practical need exists in science to narrow down lists of possibilities on the basis of objectively quantifiable plausibility.

A numerically defined Universal Plausibility Metric (UPM = ξ) has been provided in this paper. A numerical inequality of ξ < 1 establishes definitive operational falsification of any chance hypothesis (The Universal Plausibility Principle [UPP]). Both UPM and UPP pre-exist and are independent of any experimental design and data set. No low-probability plausibility assertion should survive peer-review without subjection to the UPP inequality standard of formal falsification (ξ < 1).

The use of the UPM and application of the UPP inequality to each specific UPM will promote clarity, efficiency and decisiveness in all fields of scientific methodology by allowing operational falsification of ridiculously implausible plausibility assertions. The UPP is especially important in astrobiology and all areas of life-origin research where mere theoretical possibility is often equated erroneously with plausibility. The application of The Universal Plausibility Principle (UPP) precludes the inclusion in scientific literature of wild metaphysical conjectures that conveniently ignore or illegitimately inflate probabilistic resources to beyond the limits of observational science. The UPM and UPP together prevent rapidly shrinking funding and labor resources from being wasted on preposterous notions that have no legitimate place in science. At best, notions with ξ < 1 should be considered not only operationally falsified hypotheses, but bad metaphysics on a plane equivalent to blind faith and superstition.

## Competing interests

The author declares that he has no competing interests.

## Appendix 1

2^NH ^is the "practical" number (high probability group), measured in bits, rather than the erroneous theoretical n^N ^as is usually published, of all possible biopolymeric sequences that could form, where

N = the number of loci in the string (or monomers in polymer)

n = the number of possible alphabetical symbols that could be used at each locus (4 nucleotides, 64 codons, or 20 amino acids)

H = the Shannon uncertainty at each locus

For a 100 mer biopolymeric primary structure, the number of sequence combinations is actually only 2.69 × 10^-6 ^of the theoretically possible and more intuitive measure of n^N ^sequences. The reason derives from the Shannon-McMillan-Breiman Theorem [67-70] which is explained in detail by Yockey [[71], pg 73-76].

## Appendix 2

For best estimates of the number of atoms, protons, neutrons and electrons in the universe and its astronomical subsets, see [72].

Simple arithmetic is needed for many of these calculations. For example, the mass of our galaxy is estimated to be around 10^12 ^solar masses. The mass of "normal matter" in our galaxy is around 10^11 ^solar masses. The mass of the sun is about 2 × 10^30 ^kg. The mass of our solar system is surprisingly not much more than the mass of the sun, still about 2 × 10^30 ^kg. (The Sun contains 99.85% of all the matter in the Solar System, and the planets contain only 0.136% of the mass of the solar system.) The mass of a proton or neutron is 1.7 × 10^-27 ^kg. Thus the number of protons & neutrons in our solar system is around 2 × 10^30^/1.7 × 10^-27 ^= 1.2 × 10^57^. The number of electrons is about half of that, or 0.6 × 10^57^. The number of protons, neutrons and electrons in our solar system is therefore around 1.8 × 10^57^. The number of protons, neutrons and electrons in our galaxy is around 1.8 × 10^68^. We have crudely estimated a total of 100 protons, neutrons and electrons on average per atom. All of these estimates will of course vary some through time as consensus evolves. But adjustments to ^L^Ω_A _are easily updated with absolutely no change in the Universal Plausibility Metric (UPM) equation or the Universal Plausibility Principle (UPP) inequality. Definitive operational falsification still holds when ξ < 1.
